# Innovations in acute and chronic pain biomarkers: enhancing diagnosis and personalized therapy

**DOI:** 10.1136/rapm-2024-106030

**Published:** 2025-02-05

**Authors:** Sean Mackey, Nima Aghaeepour, Brice Gaudilliere, Ming-Chih Kao, Merve Kaptan, Edward Lannon, Dario Pfyffer, Kenneth Weber

**Affiliations:** 1 Division of Pain Medicine, Stanford University School of Medicine, Stanford, California, USA; 2 Anesthesiology, Perioperative and Pain Medicine, Stanford University, Stanford, California, USA

**Keywords:** Acute Pain, CHRONIC PAIN, Multimodal Imaging, Pain Measurement, TECHNOLOGY

## Abstract

Pain affects millions worldwide, posing signiﬁcant challenges in diagnosis and treatment. Despite advances in understanding pain mechanisms, there remains a critical need for validated biomarkers to enhance diagnosis, prognostication, and personalized therapy. This review synthesizes recent advancements in identifying and validating acute and chronic pain biomarkers, including imaging, molecular, sensory, and neurophysiological approaches. We emphasize the emergence of composite, multimodal strategies that integrate psychosocial factors to improve the precision and applicability of biomarkers in chronic pain management. Neuroimaging techniques like MRI and positron emission tomography provide insights into structural and functional abnormalities related to pain, while electrophysiological methods like electroencepholography and magnetoencepholography assess dysfunctional processing in the pain neuroaxis. Molecular biomarkers, including cytokines, proteomics, and metabolites, offer diagnostic and prognostic potential, though extensive validation is needed. Integrating these biomarkers with psychosocial factors into clinical practice can revolutionize pain management by enabling personalized treatment strategies, improving patient outcomes, and potentially reducing healthcare costs. Future directions include the development of composite biomarker signatures, advances in artiﬁcial intelligence, and biomarker signature integration into clinical decision support systems. Rigorous validation and standardization efforts are also necessary to ensure these biomarkers are clinically useful. Large-scale collaborative research will be vital to driving progress in this ﬁeld and implementing these biomarkers in clinical practice. This comprehensive review highlights the potential of biomarkers to transform acute and chronic pain management, offering hope for improved diagnosis, treatment personalization, and patient outcomes.

**What is already known on this topic**

- Researchers have signiﬁcantly advanced our scientiﬁc knowledge of acute and chronic pain mechanisms. Despite advancements, a signiﬁcant need remains for validated and standardized biomarkers that enhance diagnosis, prognostication, and treatment personalization.

**What this review adds**

- Our review synthesizes recent advancements in identifying and validating biomarkers for chronic pain, including imaging, molecular, sensory, and neurophysiological. We emphasize the emergence of composite, multimodal approaches that integrate psychosocial factors to enhance the precision and applicability of biomarkers in chronic pain management. This review identiﬁes critical challenges and future research directions.

**How this review might affect research, practice or policy**

- The review underscore the importance of continued research into biomarker validation and standardization. Adopting these biomarkers in clinical practice can revolutionize chronic pain management by enabling personalized treatment strategies, improving patient outcomes, and potentially reducing healthcare costs. Additionally, the review advocates for policy changes to support large-scale collaborative research efforts in this ﬁeld.

## Introduction

An estimated 50–100 million US adults suffer from chronic pain with an annual cost of over US$500 billion per year, representing one of the most prevalent, costly, and disabling health conditions.^1 2^ According to the 2017 Global Burden of Disease Study, conditions like low back pain and headache disorders are among the leading causes of disability worldwide.^3^ The most impacted and highest-need people with chronic pain have been deﬁned as those with high-impact chronic pain (HICP) by the Health and Human Services National Pain Strategy.^4^ HICP is associated with substantially restricted work, social, and self-care activities for 6 or more months^5^ and affects over 20 million US adults.^6^ This increase and burden of pain is associated with a rise in prescription opioid use, with 275 million people globally using opioids in 2016, and 27 million developing opioid use disorders, leading to over 90 daily opioid overdose deaths in the USA.^7^ Hence, there is an urgent need to address both chronic pain and the opioid crisis.^8^

### Challenges in pain therapeutics

Current treatments for chronic pain, including pharmacological, interventional, behavioral, and surgical therapies, have limited effectiveness, evidenced by the high prevalence of chronic pain and continued opioid reliance.^9^ This is underscored by the high prevalence of chronic pain, low return to work and function, and continued reliance on opioid analgesics.^2 6 9^ Despite the approval of new non-opioid drugs for migraine by the Food and Drug Administration (FDA) (2018), a signiﬁcant gap remains in effectively treating other types of chronic pain. One major challenge is the lack of reliable biomarkers to demonstrate therapeutic target engagement, stratify patients, and predict disease progression or therapeutic response.^10^ Clinical trials often fail due to insufficient understanding of chronic pain mechanisms, poor translation of preclinical data, and large placebo responses.^11^

### Importance of biomarkers

Biomarkers as deﬁned by the FDA-National Institutes of Health (NIH) Biomarker Working Group glossary and used in this review are a “characteristic measured as an indicator of normal biological processes, pathogenic processes, or responses to a therapeutic intervention”.^12 13^ Biomarkers have shown value in many therapeutic areas, such as oncology, cardiovascular, and metabolic diseases, by predicting therapeutic responses and demonstrating proof of efficacy.^14^ For example, biomarkers like human epidermal growth factor receptor 2 in breast cancer have been associated with a fivefold reduction in clinical trial risk.^15^ AstraZeneca reported that target engagement biomarkers increased the probability of advancing projects to Phase II by 25%.^16^ Additionally, a large biomarker business intelligence analysis showed that the availability of selection or stratiﬁcation biomarkers increased the probability of success in Phase III trials by 21% and from Phase I to regulatory approval by 17.5%.^17^ Furthermore, patient stratiﬁcation biomarkers are crucial for designing clinical trials in heterogeneous conditions, like chronic pain, reducing variability and the need for large trial sizes.^18 19^

### Biomarker development and validation

The rigorous validation of biomarkers and endpoints can provide objective measures of pain, traditionally characterized by subjective self-reports.^20^ Pain is deﬁned by the International Association for the Study of Pain as “an unpleasant sensory and emotional experience associated with actual or potential tissue damage”.^21^ Current pain assessments rely on rating scales and symptom-based questionnaires, which are inﬂuenced by contextual factors and are only moderately reliable despite intensive training programs.^22^ Validated biomarkers, such as neuroimaging and neurophysiological measurements (positron emission tomography (PET), MRI, electroencepholography (EEG), quantitative sensory testing (QST)), genetic and genomic analysis, can complement self-reports by differentiating mechanisms and etiologies of various chronic pain conditions.^23 24^

### Aims and scope of this review

This article aims to review the different types of potential objective biomarkers for pain (with a focus on chronic pain), their clinical and research applications, and their limitations. We describe the “language” of biomarkers using an accepted FDA framework. We discuss advances in biomarker development including the use of advanced informatics and the call for composite, multimodal biomarkers. Finally, we discuss some challenges and future directions in biomarker discovery, validation, and implementation in clinical care using clinical decision support tools.

## Discussion

### Definitions and types of biomarkers for chronic pain

Pain is a multifaceted physiological and psychological phenomenon, presenting challenges in both research and clinical treatment due to its subjective nature and individual variability in perception. The gold standard and primary method for assessing pain is subjective reporting, such as pain rating scales. However, in situations where self-reporting is not feasible, such as with very young, elderly, inﬁrm, or unconscious patients, objective biomarkers are crucial.

### Biomarker types and their applications

**Figure 1:**
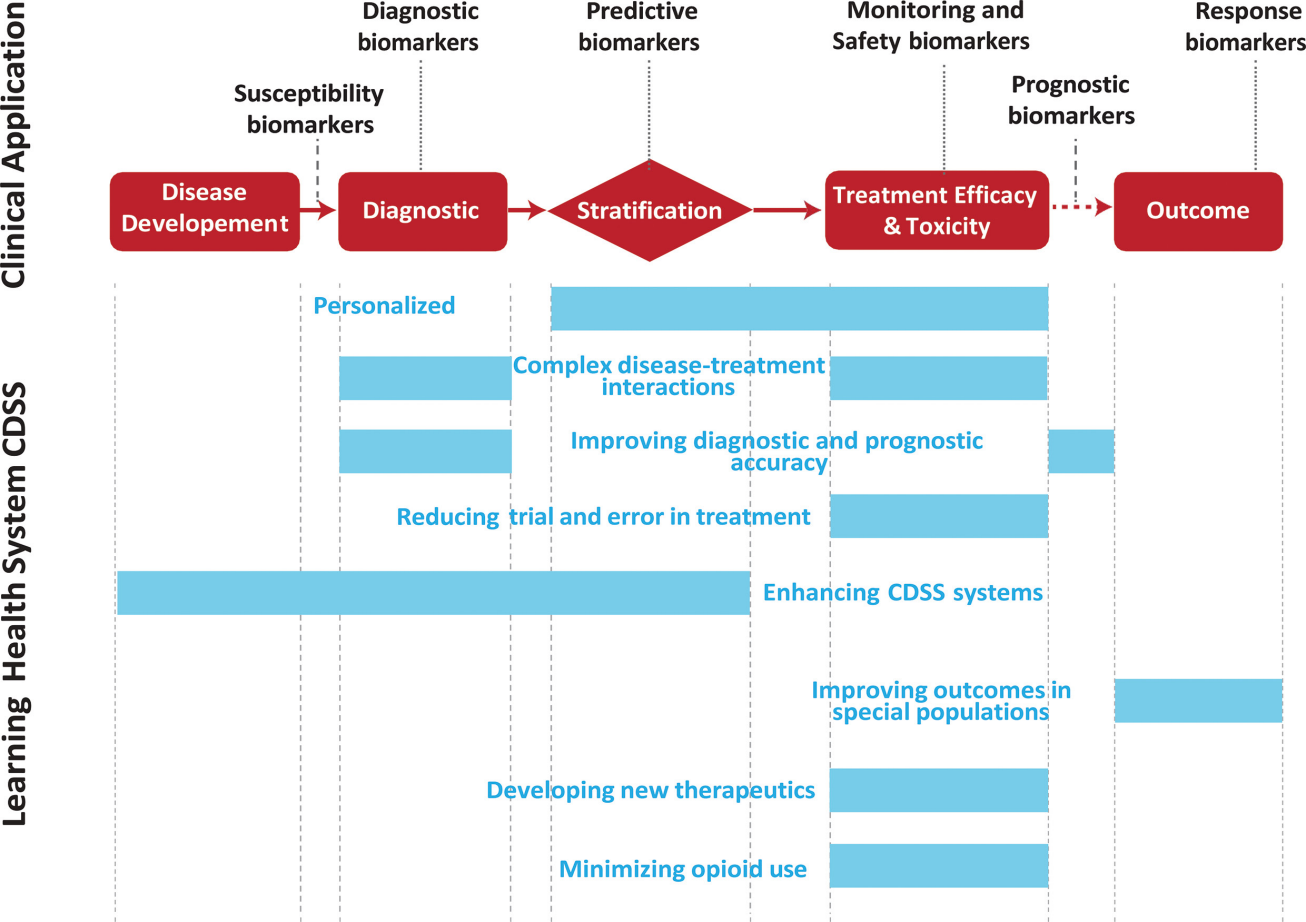
CDSS, clinical decision support system.

To advance the understanding and treatment of pain, it is essential to clearly deﬁne the different biomarker types. These biomarker types include the following (with a focus on neuroimaging biomarkers; figure 1a):

1. **Diagnostic biomarkers**: These detect or conﬁrm the presence of a condition or identify individuals within a speciﬁc subtype of a condition. For example, research has demonstrated the use of brain activity patterns to distinguish the presence and intensity of acute experimental pain, and ongoing studies are extending these methodologies to various chronic pain conditions.
2. **Prognostic biomarkers**: These indicate the likelihood of a future clinical event, disease recurrence, or progression. Studies have shown that brain connectivity patterns can predict the persistence of pain and could be used to select patients at high risk for exacerbation for targeted clinical trials.
3. **Susceptibility/risk biomarkers**: These are associated with the risk of developing a condition. While current research in neuroimaging has not yet identiﬁed risk biomarkers for chronic pain, large-scale trials are underway to identify such biomarkers, particularly in post-surgical or injury-related pain development.
4. **Predictive biomarkers**: These identify individuals likely to respond to a speciﬁc treatment. Examples from psychiatry, such as using amygdala reactivity to predict antidepressant response, illustrate the potential for similar applications in pain management. Neuroimaging could play a crucial role in predicting which patients will respond favorably to treatments like opioids or non-opioid analgesics.
5. **Monitoring biomarkers**: These are used to assess the status or extent of a condition over time, providing evidence of treatment efficacy or adverse effects. They could be instrumental in monitoring the progression of pain or opioid use postsurgery or injury
6. **Pharmacodynamic/response biomarkers**: These biomarkers change in response to a medical product or environmental agent, helping to assess clinical efficacy or safety and providing clinical decision support for treatment adjustments.
7. **Safety biomarkers**: These detect or predict adverse effects of treatments. For example, predicting which patients might experience harmful side effects from tricyclic antidepressants used for neuropathic pain could signiﬁcantly enhance treatment safety.

**Figure 2:**
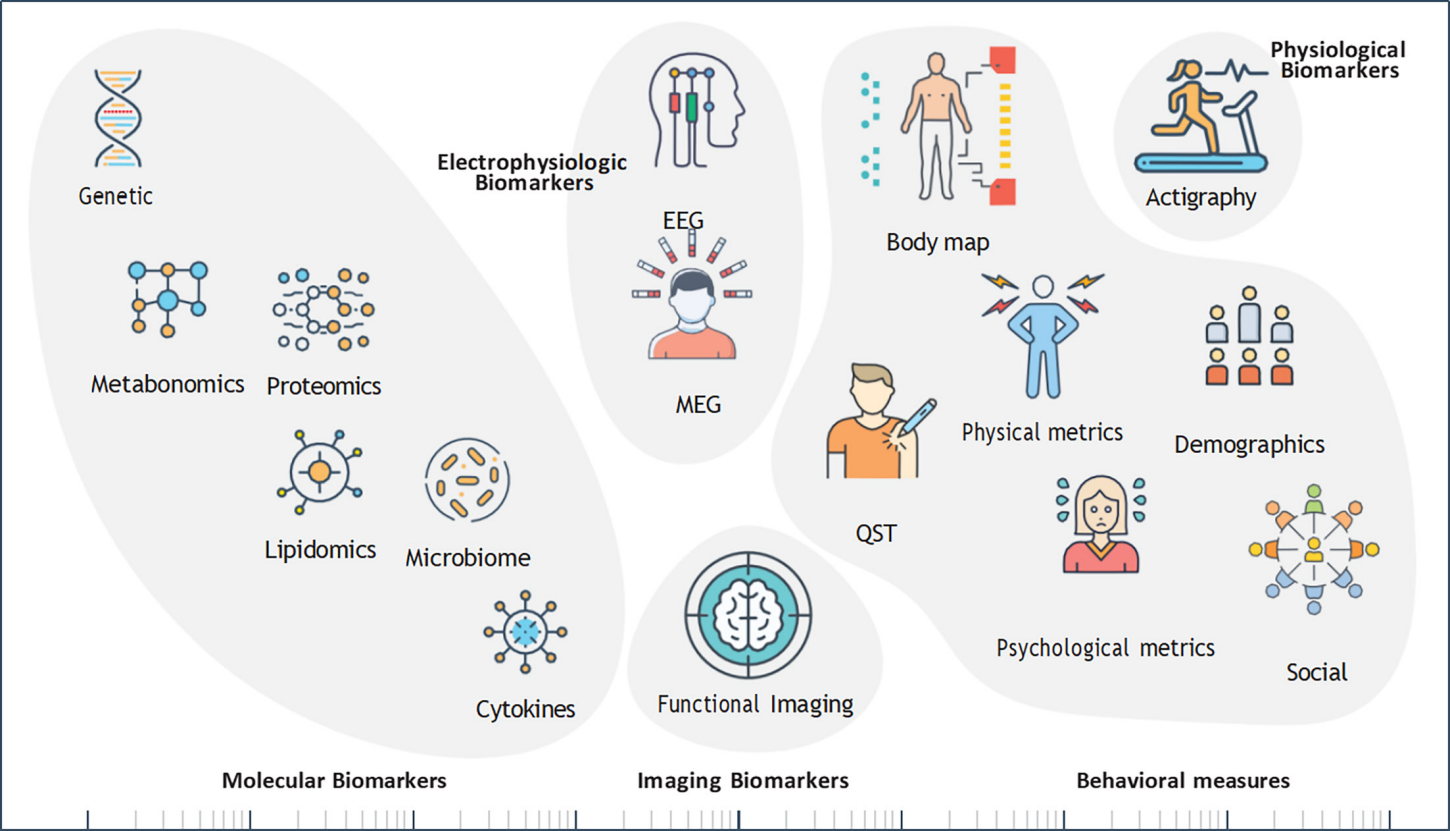
Categories of objective biomarkers and behavioral measures for pain. MEG, magnetoencephalography; QST, quantitative sensory testing.

We will brieﬂy review the different biomarkers and behavioral measures under investigation for acute and chronic pain (figure 2 and table 1).

**Table 1:** Specific Types of Biomarkers in Pain Research or Clinical Management

Biomarker type	Definition	Examples	Usage in pain research/Management
Cellular biomarkers	Measured through cell counts or activities that may be involved in pain generation or resolution	T-cell profiles in neuropathic pain, circulating immune cells in inflammatory pain	Explores immune system involvement in chronic pain and monitors inflammatory or neuropathic pain states, helping identify treatment targets
Genetic biomarkers	Variations in DNA that influence pain sensitivity or the response to pain treatments	COMT polymorphisms, OPRM1 gene variants, SCN9A mutations (linked to pain sensitivity)	Used in personalized pain treatment, identifying patients more likely to experience heightened pain or respond to specific treatments, such as opioids or NSAIDs
Proteomic biomarkers	Measures proteins that are altered in pain states or following pain treatment	C-reactive protein (CRP), inflammatory cytokines (IL-6, TNF-α), nerve growth factor (NGF)	Identifies proteins that reflect chronic pain states or treatment responses, used in the development of targeted pain therapies or diagnostics for inflammation-related pain
Metabolomic biomarkers	Metabolic products associated with pain states or treatment responses	Lactate levels (linked to muscle pain), lipid profiles in neuropathic pain	Helps understand metabolic changes in pain disorders and monitor responses to treatments, including in conditions like fibromyalgia and musculoskeletal pain
Microbiome biomarkers	Analyzes microbial compositions to identify links between gut health and chronic pain	Gut flora diversity in chronic pain, dysbiosis in fibromyalgia patients	Explores the gut-brain axis in pain conditions, indicating potential treatment strategies based on microbiome profiles
Epigenetic biomarkers	Modifications in DNA or proteins that regulate gene expression, linked to pain susceptibility or chronicity	DNA methylation in chronic pain patients, histone modifications in neuropathic pain	Investigates long-term changes in gene regulation in chronic pain, offering potential for prognosing and understanding persistent pain and finding epigenetic targets for therapy
Neurochemical biomarkers	Measures levels of neurotransmitters or neuropeptides involved in pain processing	Glutamate levels (for central sensitization), serotonin levels (in pain modulation), substance P	Explores the role of neurotransmitters in chronic pain, helping identify targets for treatment in conditions like fibromyalgia or chronic low back pain
Inflammatory biomarkers	Reflects levels of inflammation in the body, often linked to pain conditions like arthritis or fibromyalgia	IL-6, TNF-α, C-reactive protein (CRP), prostaglandins	Monitors the extent of inflammation and its relation to pain states, aiding in diagnosing and monitoring conditions such as osteoarthritis, rheumatoid arthritis, and inflammatory back pain
Imaging biomarkers	Detected through imaging technologies to assess functional, structural or chemical brain changes related to pain	fMRI (functional connectivity in pain networks), diffusion tensor imaging (DTI), PET scans (mu-opioid receptor availability)	Used to study central sensitization, CNS changes in chronic pain, effects of analgesics, as well as monitor pain-related neuroplasticity in clinical trials
Electrophysiological biomarkers	Measures electrical activity in the brain or nervous system to assess pain processing and modulation	EEG (Event-related potentials, cortical activity), MEG (magnetoencephalography for brain responses to pain stimuli), microneurography	Used to investigate altered nerve traffic and/or brain activity in chronic pain, assess pain modulation, and explore the neural mechanisms underlying pain perception and response to treatment
Behavioral markers and biomarkers	Objective markers of behavior linked to pain experiences	Actigraphy (measuring movement), pain facial expression algorithms (for facial pain recognition)	Tracks changes in pain behaviors over time, providing an objective assessment of pain-related disability or function in clinical trials and real-world settings.

### Molecular biomarkers

‘Omic approaches provide biological markers from readily accessible body compartments, including blood and stool, are relatively low cost,^25^ and show signiﬁcant diagnostic and prognostic potential for acute and chronic pain. Blood-based biomarkers are particularly promising for revealing pathophysiological mechanisms due to the ease of analysis, minimal invasiveness, and low cost. However, transitioning from exploratory data to clinically useful biomarkers will require extensive validation in large, diverse patient populations. Critical questions include whether omic signatures from accessible biospecimens (blood, urine, cerebrospinal fluid, stool) reﬂect relevant biology and which pain conditions (inﬂammatory, neuropathic) would beneﬁt from such proﬁling.

- **Cytokines**: Signiﬁcant research links cytokines, somatic problems, and musculoskeletal pain conditions.^26^,^30^ Serum cytokine levels correlate with duration of symptoms in back pain,^30^ differentiate types of back pain,^29 31^ and reveal signatures in ﬁbromyalgia.^32^
- **Metabolomics**: Metabolites are the ﬁnal downstream products of translation and, thus, are close to a studied phenotype.^33^ Several studies have characterized the role of serum metabolomics in pain.^34^,^36^ Combined with the gut microbiome (below), the serum metabolome revealed altered glutamate metabolism in ﬁbromyalgia.^34^ Studies suggest that fatigue is metabolically distinct from widespread chronic pain,^35^ and serum ornithine is associated with persistent musculoskeletal pain.^37^ Finally, a multiomics study of frailty and musculoskeletal pain identiﬁed 51 associated metabolites.^38^
- **Proteomics**: Considering the signiﬁcant cross-talk between the immune and nervous systems, systemic proteomic and cell-based signatures may mirror biological aspects of pain initiation and maintenance.^39^,^41^ Thus, proteomics shows promise for characterizing the biology of chronic pain and developing pain biomarkers.^42^ Advances in proteomic platforms, measuring over 1000 proteins, have identiﬁed predictive signatures in cardiovascular and fetomaternal medicine and immune signatures in peripheral blood have predicted the resolution of postsurgical pain.^43^,^46^
- **Lipidomics**: Lipids are vital for cellular functions such as forming cell barriers, signaling, and storing energy, and they have been suggested to play a role in pain processing and resolution. A notable example is a study integrating lipidomic measurements with transcriptomic proﬁling of lipid biosynthetic enzymes in nociceptive circuits.^47^ Lipid metabolites were associated with persistent postsurgical pain following surgery in young endometriosis patients.^48^
- **Microbiome**: The composition and health of the gut microbiome affects many chronic conditions including pain.^49^,^51^ The gut microbiome has been implicated in visceral pain,^52^,^55^ ﬁbromyalgia,^34 36 56^ interstitial cystitis,^57^ and musculoskeletal pain.^58^,^60^ Recently, researchers have called for integrating the microbiome with neuroimaging to characterize the brain-gut interaction in pain.^49^
- **Genetic**: Over the past 20 years, research has focused on uncovering the genetic underpinnings of pain. Chronic pain is signiﬁcantly heritable, with genetic factors accounting for 25–50% of the variance in pain susceptibility. Studies have identiﬁed several genes involved in pain pathways, such as COMT, OPRM1, and KCNS1. For example, genetic polymorphisms in the (*COMT*) gene are associated with the transition from acute to chronic back pain,^61^ and several chronic pain conditions, including ﬁbromyalgia, temporomandibular joint disorder,^62^ migraine, other chronic pain conditions, and persistent chronic persistent surgical pain.^63^ OPRM1 and KCNS1 are similarly linked to various chronic pain conditions and postsurgical pain. Recent genome-wide association studies have discovered numerous genetic variants associated with different pain phenotypes, though none overlap with previously identiﬁed candidate genes.^64^ These ﬁndings suggest that chronic pain is polygenic, involving many genes with small effects.

In conclusion, omic-based biomarkers are promising to enhance our understanding of pain mechanisms and improve patient management. As with the neuroimaging and electrophysiological studies noted below, substantial research and validation are required to translate these exploratory ﬁndings into clinically useful tools. Additionally, a single ‘omic biomarker is unlikely to have discriminative power with clinical utility. Promising technologies and analytical frameworks that integrate multiple ‘omic biomarkers may be vital for developing useful signatures in acute and chronic pain.^65^ One such technology is Cytometry by Time-of-Flight (CyTOF). CyTOF is a powerful mass cytometry technique for high-dimensional and high-throughput single-cell analysis. CyTOF allows for the simultaneous quantiﬁcation of multiple cellular components, providing signiﬁcant insights into cellular functions and phenotypes.^66^ This high-content omic proﬁling will require sophisticated machine-learning approaches to develop predictive signatures with clinical utility. One such promising approach is Stabl, a recently developed machine learning approach for high-dimensional multi-omic data yielding reliable predictive biomarkers^67^

### Imaging biomarkers

Neuroimaging reveals both structural, chemical and functional abnormalities in pathways related to nociception and other functions. Techniques like ultrasound, MRI, PET, and CT are widely used to detect structural pathologies in both peripheral and central nervous system tissues. Common structural imaging techniques include anatomical MRI to measure cortical thickness, volume, and gray matter density, and diffusion-weighted imaging to assess white matter integrity and pathways. Magnetic resonance spectroscopy can be used to measure resting or task-evoked levels of metabolites such as glutamate and gamma-aminobutyric acid (GABA),^68^ although its spatial and temporal resolution is low. Newer methods such as magnetic resonance elastography and hyperspectral imaging allow for non-invasive detection of cellular and biochemical changes in tissues.^69^,^71^

Functional imaging can be achieved using PET, functional near-infrared spectroscopy (fNIRS), and functional MRI (fMRI), all of which are able to measure functional changes during rest, an experimental task, or sensory stimulation. PET examines the metabolism and neurochemistry of numerous neurotransmitters and neuropeptides, whereas fNIRS and fMRI quantify central nervous system hemodynamics—an indirect measure of neural activity. fNIRS uses the differential optical properties of hemoglobin at the near-infrared spectrum, while fMRI is based on the blood oxygenation-level dependent contrast due to the differential magnetic properties of oxygenated and deoxygenated hemoglobin.

Central neuroimaging techniques, such as fMRI, enable researchers to characterize the function of the brain, brainstem and spinal cord. These techniques have reshaped our understanding of the magniﬁcation, persistence, alleviation of pain, and targets for treatment.^72 73^ Additionally, neuroimaging has yielded insights into the modulatory role of anxiety, fear, catastrophizing, depression, placebo and individual differences in pain.^74^,^76^ Preclinical imaging studies have highlighted potential pain mechanisms in various nervous system pathways, including sensitization of nociceptive neurons and alterations in frontostriatal pathways.^77^ While most of the neuroimaging studies have focused on the brain, recent techniques have been developed to image the spinal cord and even the entire central nervous system.^78^,^84^

Researchers have extended the mechanistic insights from neuroimaging to develop objective biomarkers of pain. Current research focuses on developing and validating imaging-based biomarker for evoked pain sensitivity,^85^,^89^ and as diagnostic,^8790^,^94^ prognostic,^95^,^97^ predictive,^98^,^102^ or response biomarkers for clinical pain.^87^ Similarly, structural MRI-based metrics are used to develop diagnostic^103^,^107^ and prognostic^104^ pain biomarkers. Combining fMRI-based biomarkers with clinical scores was shown to improve the classiﬁcation of chronic pain conditions such as migraine^108^ and predict future chronic pain in mild traumatic brain injury patients.^109^ All these highlight the need for multimodal approaches for pain biomarker development (discussed in “Composite biomarker signature for pain” section below).^110^

### Electrophysiological biomarkers

Electrophysiological methods can assess dysfunctional processing at multiple levels of the pain neuroaxis. Microneurography, which records peripheral nerve activation, can measure abnormalities of peripheral nerves found in pain conditions such as spontaneous activation of somatosensory afferents, including Aδ-fiber and C-ﬁber nociceptors.^111^ To measure brain activity, non-invasive methods such as EEG and magnetoencephalography (MEG) measure synchronous postsynaptic potentials of pyramidal cell networks from brain systems associated with pain.^112 113^

MEG and EEG measure distinct components of the electromagnetic ﬁeld, each with their own advantages. MEG is well-suited for source-level analysis due to the minimal disturbance of the magnetic ﬁeld by tissue. As with MRI, MEG is limited by large expensive equipment. EEG is characterized by its affordability and ease of use in clinical settings.^114^ The two primary approaches are event-related and resting-state, corresponding to stimulus-evoked and spontaneous pain, respectively.

Evoked gamma-band activation (30–100 Hz) has been linked to noxious stimuli in both rodents and humans produced by GABAergic interneurons with both local and distal reach.^115^ Gamma band oscillations are unaffected by stimulus salience making them useful markers of the pain system. Increased resting state Theta (4–8 Hz), indicating nociceptive and nociplastic pain, is modulated by pharmacological interventions and neuromodulation.^113 116^ A slowing of peak alpha frequency has recently gained attention as a marker of pain sensitivity in individuals without chronic pain. However, its speciﬁcity to pain, and not overall hypersensitivity, is unknown. Increased theta and a slowing of peak alpha frequency have been associated with thalamocortical dysrhythmia, characterized by hypersensitization of thalamocortical relay neurons leading to excessive cortical inhibition.^113 114^ While these electrophysiologic insights have advanced our understanding of acute and chronic pain, further research is needed to determine which will have clinical utility as a validated biomarker.

### Physiological biomarkers

#### Actigraphy for function and sleep

Actigraphy, capturing three-dimensional accelerometry data, has been widely used to monitor physical movement and activity–rest cycles. It provides detailed information about movement levels day and night, which can be crucial for predicting potential diseases and personalizing medical services for individuals with acute and chronic pain.^117^ Actigraphy is also extensively used in sleep medicine to estimate sleep parameters over extended periods, often in the patient's natural environment. The device tracks movements to analyze when a person is asleep and awake, providing estimates for sleep latency, total sleep time, wake after sleep onset, and sleep efficiency.^118^ Despite its advantages, actigraphy has limitations. It does not accurately measure sleep architecture (stages of sleep) and can sometimes misclassify wakefulness as sleep or vice versa, especially in patients with excessive movements during sleep (as in those in pain) or those who lie still while awake. Furthermore, subjective sleep quality is poorly associated with actigraphy.^119^ Finally, the accuracy of actigraphy can be affected by factors such as the type of device used and the algorithms applied to the data. Despite these limitations, the ease and low cost of capturing these data make actigraphy an appealing potential biomarker for acute and chronic pain.

### Behavioral measures

#### Physical, psychological, social, demographic, and body map metrics

Multiple factors are associated with chronic pain and its impact, including increasing age, manual work, longer pain duration, widespreadness of pain,^120 121^ anxiety,^122^ self-efficacy,^123^ pain catastrophizing,^124^ and social isolation.^125^ Similarly, widespread pain, high functional disability, somatization, high pain intensity, previous pain episodes, and pain medication use are associated with treatment outcomes.^126^ Increasing pain intensity, pain interference, pain duration, and additional pain sites are prognostic for chronic pain.^127 128^ Integrating these demographic and psychosocial factors with biological data signiﬁcantly advances biomarker development for chronic pain. By incorporating these factors into biomarker research, we can develop more holistic models that better capture the complexities of chronic pain. For example, Gilam *et al*, used a clustering of “pain agnostic” psychosocial measures to classify chronic pain and its prognosis.^129^

While subjective behavioral measures—such as anxiety or pain catastrophizing—are not traditionally classiﬁed as biological biomarkers, they play a crucial role in pain research. Additionally, objective behavioral measures can serve as predictive behavioral markers (PBMs), offering valuable insights into treatment outcomes or risk proﬁles. Hagopian *et al*^130^ showed that PBMs can predict response to interventions, underscoring their utility in pain management. These behavioral measures complement biological biomarkers, helping capture the complex biopsychosocial nature of pain. Examples of PBMs include objective measures of sleep disturbances, substance use, medication adherence behavior, objective responses to emotional stimulation and psychological stress, and functional movement patterns. These PBMs can modify the expression of biological biomarkers, inﬂuencing both pain outcomes and treatment responses. Recognizing these behavioral and lifestyle factors as important modiﬁers in pain research ensures a more comprehensive and personalized approach to treatment. By incorporating both biological and behavioral measures, we can better understand and address the full spectrum of inﬂuences of pain, particularly for vulnerable populations who may experience higher variability in these factors

#### Quantitative sensory testing

QST reveals mechanistic information via standardized behavioral testing for pain phenotyping.^131^,^134^ Large multinational efforts in Europe are integrating QST in protocolized characterization of patients with neuropathic pain.^135^ QST modalities are generally classiﬁed as static or dynamic measures.^136^ Studies have shown that static QST measures such as pressure pain threshold are diagnostic biomarkers of temporomandibular joint pain.^137^ Dynamic QST measures—such as temporal summation^138^ and conditioned pain modulation—are purported to reveal information on central pain processing.^139^ Evidence suggests that temporal summation is a prognostic biomarker of knee pain intensity,^140^ and a predictive biomarker of post-surgical pain after knee replacement^141^ and thoracotomy.^142^

### Advances in biomarker development

#### Technological innovations

Recent technological advances have signiﬁcantly enhanced the discovery and validation of biomarkers for chronic pain. Machine learning (ML) and artiﬁcial intelligence (AI) are at the forefront of these innovations, offering powerful tools for analyzing complex, multidimensional data. By leveraging these technologies, researchers can identify patterns and correlations within large datasets that would be impossible to discern manually. These tools are particularly useful for integrating various types of data, such as imaging, proteomic, and genetic data, to develop more comprehensive and accurate biomarkers.

AI-driven approaches also improve the ability to predict individual responses to pain treatments and therapeutic target discovery, thereby facilitating personalized medicine.^143 144^

AI and ML have the potential to advance biomarker discovery signiﬁcantly, but they also introduce challenges that must be addressed. One major concern is overﬁtting, where models capture noise or spurious correlations, leading to “data hallucinations” and the identiﬁcation of false biomarkers. To mitigate this, it is essential to apply rigorous validation strategies, including independent datasets and cross-validation techniques, to ensure that the biomarkers identiﬁed are accurate and generalizable. Additionally, many AI/ML models lack transparency, operating as “black boxes” and making it difficult to interpret how speciﬁc biomarkers relate to biological mechanisms. Developing more interpretable models or incorporating explainability methods is crucial to translating AI-driven discoveries into clinical practice.

Bias in training data is another critical challenge, particularly when datasets under-represent marginalized populations. This can result in models that produce biased or inaccurate results, exacerbating health disparities when applied in clinical settings. Biomarkers identiﬁed from homogeneous datasets may not generalize well to diverse populations, leading to inequitable healthcare outcomes. To address this, it is essential to use diverse and representative datasets and to implement bias detection and correction methods to ensure that the resulting biomarkers are broadly applicable.

Finally, the issue of incomplete or noisy data adds another layer of complexity. Clinical datasets often miss critical information, and AI models may produce unreliable results if these gaps are not properly managed. While data imputation can help address missing data, these methods may introduce uncertainty or reinforce existing biases. Handling incomplete data carefully and ensuring the integration of domain expertize throughout the AI/ML process will help reduce the risk of false conclusions regarding biomarkers and therapeutic targets.

#### 
Composite biomarker signature for pain

The biopsychosocial model of pain has proven to be the most heuristic approach to understanding and managing pain. This model assumes that pain and its disability is a complex and dynamic interaction among physiological, psychological, and social factors that can maintain and amplify pain and disability.^145^ An isolated approach to assessing pain leads to models with limited predictive power. Indeed, the NIH convened a workshop of 29 national experts (including authors of this review) on the “Discovery and Validation of Biomarkers to Develop Non-Addictive Therapeutics for Pain”. A key ﬁnding was that a more comprehensive and composite assessment of a chronic pain signature may lead to new and improved treatments.^146^ Similarly, a Neuron Perspective^110^ noted that one biomarker is highly unlikely to capture pain, and a ‘‘composite pain biomarker signature’’ is more promising. Therefore, combining neuroimaging data with non-neuroimaging data, such as behavioral, omic, and physiologic information, could improve the sensitivity and speciﬁcity of pain biomarkers. Integrating multiple data sources into a multimodal biomarker approach is likely necessary to capture the complete variance in pain models and improve clinical utility.

A current multisite observational study aims to develop composite biomarkers to predict chronic pain development after surgical intervention using neuroimaging, omics, psychophysical, psychological, and behavioral measures.^147^ Another NIH-funded, multisite study is developing composite prognostic biomarkers in children with musculoskeletal pain.^148^ The authors of this review are engaged in an NIH-funded study to develop composite, diagnostic, and prognostic biomarkers for high-impact chronic pain.

### Ethical considerations in the use of pain biomarkers

Integrating biomarkers into pain research and clinical care introduces several ethical challenges. One of the primary concerns is the potential invalidation of a patient’s pain experience when speciﬁc biomarkers are absent. Pain is subjective, and relying solely on biomarkers risks dismissing or undertreating individuals who do not exhibit measurable biological markers. It is essential to treat biomarkers as complementary to self-reports, rather than as deﬁnitive indicators of pain. Furthermore, under-representing vulnerable populations in biomarker research can result in inaccurate diagnoses and exacerbate health disparities. To ensure equity, biomarker studies must include diverse populations, avoiding the exclusion of groups historically underserved in healthcare.

Another major concern is the improper use of pain biomarkers by employers, insurers, and the legal system. Biomarkers that indicate pain sensitivity or predisposition to chronic pain may lead to discrimination, such as denying employment or insurance coverage. This raises ethical questions about the fairness of using biomarkers to make decisions in non-medical contexts. Additionally, resource allocation could become an issue, with expensive treatments being deﬂected away from patients who lack speciﬁc biomarkers. Medical privacy and data security are also paramount, as the misuse or breach of sensitive biomarker data could signiﬁcantly harm patients. Protections must be implemented to ensure that biomarkers are used ethically, safeguarding patient rights and avoiding misuse for ﬁnancial, legal, or social discrimination.

In addition to advancing diagnostic precision, pain biomarkers hold promise for addressing disparities in pain management across vulnerable populations. Neuroimaging biomarkers, for example, provide objective insights into how pain is processed in the brain, helping to validate the pain experiences of individuals who may otherwise be dismissed. This is particularly important for groups historically underserved in healthcare, such as ethnic minorities, the elderly, the very young, and women, who often face biases in pain assessment and treatment. By offering biological evidence of pain, these biomarkers can counteract the perception that “pain is in the head” in a dismissive sense, reframing it as a neurologically-based and measurable phenomenon. Biomarkers not only offer the potential to improve personalized treatment for these populations but also to bridge the gap between subjective pain reports and objective clinical validation, fostering greater trust between patients and providers.

### Integrating biomarker signatures into clinical practice

Integrating biomarker signatures into clinical decision support tools is paramount for enhancing clinical practice’s precision and efficacy. Biomarker signatures offer a nuanced understanding of disease mechanisms and patient responses, enabling clinicians to tailor interventions more accurately to individual patient proﬁles. By embedding these signatures within clinical decision support systems, clinicians can access real-time, evidence-based recommendations that consider the unique biological characteristics of each patient. This integration improves diagnostic accuracy and treatment outcomes and facilitates the early detection of disease progression and therapeutic efficacy monitoring. Moreover, using biomarker-driven decision tools can reduce variability in clinical practice, ensuring more standardized and effective care across diverse patient populations. As healthcare continues to move toward personalized medicine, incorporating biomarker signatures into clinical decision support systems is essential for optimizing patient outcomes and advancing the quality of care.

#### Cost and practicality

While neuroimaging and molecular biomarkers hold signiﬁcant promise for advancing precision pain diagnosis and management, several challenges remain regarding their cost and practicality. Technologies like fMRI and advanced molecular assays such as CyTOF are resource-intensive, requiring specialized equipment, trained personnel, and considerable infrastructure, which limits their accessibility and practicality in routine clinical settings. These barriers are particularly pronounced in low-resource environments and can exacerbate healthcare disparities if not addressed. In contrast, other biomarkers, such as simpler cytokine measurements, patient-reported outcomes, and demographic measures, actigraphy or routine blood tests, are more practical and cost-effective, making them feasible for broader clinical implementation. For biomarker integration to be successful across diverse clinical settings, it will be essential to balance the use of advanced, complex biomarkers with more accessible, scalable options. This approach ensures that the beneﬁts of precision pain management are available to a broader patient population without disproportionately favoring well-resourced healthcare systems.

Integrating biomarker signatures into clinical decision support system (CDSS) tools is crucial for enhancing the precision and efficacy of clinical decision-making. These tools are best implemented through what the National Academy of Medicine has called the learning health system (LHS).^149^,^151^ An LHS is a healthcare framework that continuously and systematically integrates data and evidence from clinical practice and research into the healthcare process, enabling real-time learning and improvement in patient care. Some key uses of biomarker integration into clinical decision support tools and LHSs include (figure 1):

1. **Enhanced personalization of therapy**:

Biomarker signatures can facilitate personalized therapy for pain by providing clinicians with detailed, patient-speciﬁc information. This approach allows for tailoring therapeutic strategies to individual patients’ proﬁles, thereby improving treatment outcomes.

1. **Complex disease-treatment interaction**:

Using biomarker signatures can help clinicians navigate the complex interactions between disease pathophysiology and treatment mechanisms. By accounting for the diversity in clinical phenotypes, these signatures can provide a more comprehensive understanding of the disease state, enabling more accurate and informed decision-making.

1. **Improving diagnostic and prognostic accuracy**:

Biomarker signatures can enhance chronic pain diagnostic and prognostic accuracy by offering a more nuanced view of the patient’s condition. For example, in precision pain medicine, the integration of multimodal biomarkers helps better understand disease progression or response to treatment, thereby guiding clinicians in making more accurate diagnoses and prognoses.

1. **Reducing trial and error in treatment**:

Incorporating biomarker signatures into CDSS can reduce the frustrating trial-and-error approach often seen in clinical practice. By leveraging these signatures, clinicians can make more informed decisions about which treatments are likely effective, thereby minimizing the risk of adverse reactions and improving patient outcomes. Patients will get the beneﬁt of an earlier time to effective treatment and potential impact on reducing chronicity and disability of pain.

1. **Enhancing clinical decision support systems**:

Integrating biomarker signatures into CDSS can enhance the overall effectiveness of these systems. By providing timely and intelligent processing of patient-speciﬁc data, CDSS can support clinicians in making more accurate and informed decisions, ultimately improving patient care and reducing healthcare costs.

1. **Development of new therapeutics**:

Biomarkers can help identify new molecular targets for pain management, driving the development of novel analgesics and therapies. Additionally, incorporating biomarkers in clinical trials can enhance the precision of efficacy assessments, stratify patients more effectively, and reduce heterogeneity in study populations.

1. **Minimizing opioid use and enhancing opioid stewardship**:

Biomarker signatures can help predict susceptibility to opioid addiction and misuse, informing opioid prescribing practices. These signatures can support the development of tapering protocols by identifying patients likely to beneﬁt from speciﬁc tapering strategies and those at risk of withdrawal symptoms or relapse. Additionally, biomarker signatures can help identify those patients who are most likely to respond to short-term or long-term use of opioids.

1. **Improving outcomes in special populations**:

Biomarker signatures can help tailor pain management strategies for children, who often present unique challenges in pain assessment and treatment. In elderly patients, biomarkers can inform pain management while accounting for comorbidities and polypharmacy issues.

1. **Integrating multidisciplinary pain management approaches**:

Biomarker signatures can be integrated with psychosocial assessments to provide a comprehensive understanding of pain mechanisms, facilitating multidisciplinary approaches to pain management. These signatures can guide rehabilitation protocols by identifying patients who might beneﬁt from speciﬁc physical or psychological therapies to enhance recovery and pain resolution.

## Summary and future directions

In summary, biomarkers are essential in advancing acute and chronic pain management by guiding the development of personalized treatment strategies. Biomarkers can indicate therapeutic target engagement, predict therapeutic response, improve clinical trial designs by stratifying patients, and monitor safety and efficacy.^12 152^ While there are challenges in adopting these biomarkers, such as the need for rigorous validation and the complexity of chronic pain mechanisms, their potential beneﬁts in improving diagnosis, prognosis, and treatment are substantial. Continued research and investment in this area are essential for realizing the full potential of biomarkers in acute and chronic pain management. Integrating AI, ML, objective biomarkers and patient-reported outcomes can transform pain management research by enhancing disease diagnosis and treatment approaches. This effort will require a multidisciplinary approach, with collaboration among researchers, pain management specialists, other healthcare professionals, and people with lived experience with pain. Finally, integrating biomarker signatures into clinical decision-support tools is essential for advancing personalized medicine, improving diagnostic and prognostic accuracy, and enhancing the overall quality of clinical decision-making. This approach can potentially revolutionize healthcare by providing clinicians with the tools necessary to make more informed, patient-speciﬁc decisions.

## Data Availability

No data are available.
